# Supplementary material for: Comparative Study of the Potentially Toxic Elements and Essential Microelements in Honey Depending on the Geographic Origin
Source: Molecules. 2022 Aug 26;27(17):5474. doi: 10.3390/molecules27175474 (PMC9457584; doi:10.3390/molecules27175474)
Supplement: Supplementary file 1 [file molecules-27-05474-s001.zip › molecules-1832184-supplementary.pdf]

Supplementary Table S1. The detailed composition in PTEs of each analysed samples including number of sample. origin and type of honey (mean contents given in unit μg/kg; n = 3)

Sample	Origin Country/Province	Type	As 75	Be 9	Co 59	Cr 52	Cu 63	Fe 56	Mg 24	Mn 55	Mo 98	Ni 60	Sb 121	V 51	Zn 66
01	Poland/Lesser Poland	buckwheat	< LOD	< LOD	0.976	0.1596	262.0	1112	4380	1258.0	0.2440	1.666	0430	0426	614000
02	Poland/Holy Cross Province	buckwheat	< LOD	< LOD	0.384	< LOD	296.0	1024	6820	1544.0	0.1306	4.600	0006	0558	576000
03	Poland/Warmian-Masurian	buckwheat	0.4300	< LOD	0.328	3.760	248.0	1256	5280	672.0	0.3500	5.780	0.2140	0.1184	502000
04	Poland/Lesser Poland	multifloral	< LOD	< LOD	0.294	0.131	11.42	802	5080	145.2	0566	1.352	0138	0160	414000
05	Poland/Masovia	multifloral	< LOD	< LOD	0.228	0.136	199.4	784	7020	456.0	0.1066	360.1	0000	0296	450000
06	Poland/Lesser Poland	honeydew	0.2400	< LOD	4.900	0.428	402.0	1160	14980	956.0	0.6120	167.2	0.1206	0.2740	570000
07	Poland/Lesser Poland	honeydew	0.2640	< LOD	7.280	0.416	464.0	1056	14060	844.0	0.8160	266.0	0.1094	0.3120	482000
08	Poland/Kuyavia-Pomerania	phacelia	< LOD	< LOD	0.832	0.268	230.0	866	10580	504.0	0.2800	7.260	0000	0802	534000
09	Poland/Lesser Poland	rapeseed	< LOD	< LOD	0.228	0.782	6.48	632	4480	49.2	0138	1.828	0000	0226	252000
10	Poland/Lesser Poland	linden	< LOD	< LOD	0.372	0.320	19.68	756	4900	264.0	0.1612	2.860	0000	0718	340000
11	Bulgaria	sunflower	< LOD	< LOD	1.480	0.356	177.8	844	6260	120.2	0.1378	2.940	0000	0736	428000
12	Poland/Lesser Poland	goldenrod	< LOD	< LOD	0.422	0.187	11.4	606	4080	73.4	0.1190	1.636	0000	0240	258000
13	Poland/Silesia	goldenrod	0.1812	< LOD	2.800	0.145	224.0	778	9740	670.0	0.1876	20.8	0000	0964	384000
14	Poland/Łódź	goldenrod	< LOD	< LOD	0.262	0.280	180.6	798	6940	132.8	0.1642	1.370	0270	0.1072	296000
15	Poland/Łódź	goldenrod	< LOD	< LOD	0.846	0.560	14.21	788	16080	195.0	0.4280	6.460	0000	0.3520	300000
16	Poland/Kuyavia-Pomerania	rapeseed	< LOD	< LOD	0.250	< LOD	5.18	576	4240	95.6	0532	0.414	0000	0170	19.2200

17	Poland/Kuyavia-Pomerania	rapeseed	< LOD	< LOD	0.428	0.160	14.72	724	6560	82.2	0.2180	0.958	0000	0544	282000
18	Poland/Lower Silesia	buckwheat	< LOD	< LOD	0.204	0.101	426.0	900	4640	2100.0	0.1162	2.180	0000	0362	506000
19	Poland/Lublin	multifloral	< LOD	< LOD	0.496	0.145	224.0	842	6340	458.0	0.2880	1.900	0000	0.1460	368000
20	Poland/Greater Poland	sunflower	< LOD	< LOD	0.534	0.394	12.52	808	6940	64.2	0578	3.720	0000	0678	342000
21	Poland/Greater Poland	malinowy	< LOD	0.758	0.376	0.644	9.43	776	6440	140.8	0.2780	1.280	0718	0130	326000
22	Portugal	rosemary	< LOD	0.101	0.248	0.110	6.51	750	3580	80.8	0.1292	0.754	0.1220	0.1002	242000
23	Portugal (Madera)	multifloral	< LOD	0.126	4.220	1.480	186.2	790	9360	284.0	0.3020	19.24	0516	0.1376	336000
24	Tasmania	bush	< LOD	0.126	2.480	0.106	12.85	692	4900	600.0	0670	1.760	0.1040	0554	276000
25	Tasmania	leather tree	< LOD	0.758	4.600	0.516	12.92	798	3780	518.0	0332	2.160	0468	0302	240000
26	Tasmania	clover	< LOD	0.884	0.482	0.114	6.48	952	3940	290.0	0.5920	1.274	0426	0.1262	282000
27	Poland/Kuyavia-Pomerania	buckwheat	< LOD	0.884	0.356	< LOD	460.0	788	5900	2080.0	0.3680	9.20	0502	0580	586000
28	Poland/Kuyavia-Pomerania	linden	0.980	0.758	0.372	0.114	19.58	860	9280	108.8	1.5480	1.892	0.2060	0.2660	348000
29	Poland/Kuyavia-Pomerania	linden	< LOD	0.101	0.462	0.108	18.68	1146	5780	73.0	0.4960	2.200	0548	0698	394000
30	Italy	multifloral	< LOD	0.758	0.420	0.384	17.98	876	6220	228.0	0782	15.400	0744	0484	354000
31	Poland/Kuyavia-Pomerania	multifloral	< LOD	0.884	0.240	< LOD	5.96	706	4340	14.5	0.1220	0.410	0516	0154	482000
32	Poland/Warmian-Masurian	acacia	< LOD	0.632	0.272	< LOD	7.64	720	3980	118.0	0114	3.3600	0430	0066	358000
33	Poland/Warmian-Masurian	linden	< LOD	0.101	0.378	< LOD	16.70	838	5120	222.0	0.1070	2400	0462	0166	440000
34	Poland/Warmian-Masurian	buckwheat	0.1292	0.101	0.420	0.320	218.00	<LOD	4880	396.0	0.1460	6200	0.2260	0648	1048000
35	Turkey	multifloral	0.4900	0.758	0.114	0.103	2.22	13.4	2860	2800	0176	0.2220	0.2540	0490	3.9000

36	Poland/Lower Silesia	acacia	< LOD	0.884	0.174	< LOD	4.84	722	3380	13.5	0252	0.9020	0408	0008	332000
37	Romania	multifloral	< LOD	0.101	0.414	0.552	9.34	1186	5860	83.0	0.1154	1.8900	0.2420	0510	318000
38	Romania	rapeseed	< LOD	0.884	0.202	< LOD	12.94	772	10140	14.7	0.1126	6.7400	0438	0188	392000
39	Romania	acacia	< LOD	0.101	0.190	0.730	5.36	714	2920	15.5	0.1118	2.5800	0444	0166	288000
40	Poland/Lublin	linden	< LOD	0.884	0.195	< LOD	364.0	904	3860	1708.0	0300	1.1180	0484	0188	372000
41	Poland/Lublin	multifloral	< LOD	0.884	0.187	< LOD	392.0	902	4200	1942.0	0238	1700	0414	0140	408000
42	Poland/Warmian-Masurian	honeydew	0.306	0.356	0.484	0.288	222.0	1070	5240	442.0	0.9020	3.4200	0468	0.1390	436000
43	Poland/Warmian-Masurian	linden	0.486	0498	0.300	0.252	230.1	820	4720	402.0	0.7360	3.7800	0396	0.2140	426000
44	Poland/Warmian-Masurian	dandelion	0.151	0.498	1.112	1.140	220.1	912	4740	94.4	5.9400	6.2400	0.1272	0.2540	584000
45	Germany	multifloral	0.632	0.214	1.600	0.538	224.1	728	4340	74.4	2000	1.3800	0.1506	0.1656	492000

Supplementary Table S2. Accuracy testing by investigation of certified material (p-Alfaalfa) for selected PTE's (n = 3)

No.	Element	Type	Value	Unit	Confidence limit for sign. level (α =0.05	Obtained value	RSD [%]
3	Cd	certified	136	ppb	130-143	141.6	3.5
5	Cr	additional without confidence values	900	ppb	-	865	4.5
6	Cu	certified	11.7	ppm	10.9-12.4	10.8	2.5
7	Fe	certified	355	ppm	337-373	348	3.1
8	Mg	certified	0.352	%	0.340-0.365	0.354	5.1
9	Mn	certified	34.2	ppm	33.1-35.4	35.2	3.3
11	Ni	certified	2.54	ppm	2.36-2.72	2.66	3.2
12	Pb	certified	1.84	ppm	1.67-2.01	1.77	2.6
15	Zn	certified	33.2	ppm	32.2-34.2	34.2	5.5
